# High-sucrose diet induces MMP-9 activation and collagen deposition via oxidative stress in Swiss mice liver

**DOI:** 10.1007/s00418-026-02523-4

**Published:** 2026-08-20

**Authors:** Mariana Alves Passos, Juliana Leticia Silva, Túlio César Rodrigues Leite, Isabela Jesus de Deus, Raianne dos Santos Baleeiro, Rafaella Dhom Ferrari Matos Gomes, Wanderson Geraldo Lima, Carla Speroni Ceron, Sílvia Paula-Gomes

**Affiliations:** 1 Laboratory of Biochemistry and Molecular Biology, Research Center in Biological Sciences, Department of Biological Sciences, Federal University of Ouro Preto, Room 248, Ouro Preto, Minas Gerais, Brazil; 2 Postgraduate Program in Biological Sciences, Research Center in Biological Sciences, Federal University of Ouro Preto, Ouro Preto, Minas Gerais, Brazil; 3 Laboratory of Experimental Nutrition, School of Nutrition, Postgraduate Program in Health and Nutrition, Federal University of Ouro Preto, Ouro Preto, Minas Gerais, Brazil; 4 Laboratory of Morphopathology, Research Center in Biological Sciences, Department of Biological Sciences, Federal University of Ouro Preto, Ouro Preto, Minas Gerais, Brazil; 5 Laboratory of Endocrine and Cardiovascular Physiology, Department of Biological Sciences, Federal University of Ouro Preto, Ouro Preto, Minas Gerais, Brazil

**Keywords:** Sucrose, Oxidative stress, Matrix metalloproteinases, Insulin signaling, Liver, AKT

## Abstract

Excessive sucrose consumption is associated with various metabolic disorders, including obesity, diabetes, and liver dysfunction. Although oxidative stress is a well-established mechanism contributing to these conditions, its effects on matrix metalloproteinase (MMP) activity in the liver remain poorly understood. This study evaluated the impact of a high-sucrose diet on oxidative stress, MMP-2 and MMP-9 activities, insulin signaling and collagen deposition in the liver of Swiss mice. Male Swiss mice (protocol no. 7,422,050,723) were divided into 2 groups with 16 animals each—control and high-sucrose diet—and were fed for 4 weeks. Liver samples were collected for histomorphological analysis of steatosis, inflammatory infiltrates, and collagen deposition, as well as for biochemical evaluation of antioxidant enzyme activity, oxidative stress markers, MMP activity, and AKT phosphorylated at serine 473. The high-sucrose diet induced metabolic imbalance, steatosis, and inflammatory infiltration, accompanied by oxidative stress in the liver, evidenced by reduced catalase activity (CAT) and glutathione levels (GSH), along with increased protein carbonyls (PCO) and thiobarbituric acid reactive substances (TBARS) levels. Additionally, MMP-9 activity and collagen deposition were higher in mice fed the high-sucrose diet. Notably, AKT phosphorylation at Ser-473 residue was reduced, indicating impaired insulin signaling. These findings suggest that a high-sucrose diet induces hepatic oxidative stress, disrupts insulin signaling, and promotes MMP-9 activation, potentially contributing to liver histopathological changes and dysfunction.

**Graphical abstract:**

**Figure:**
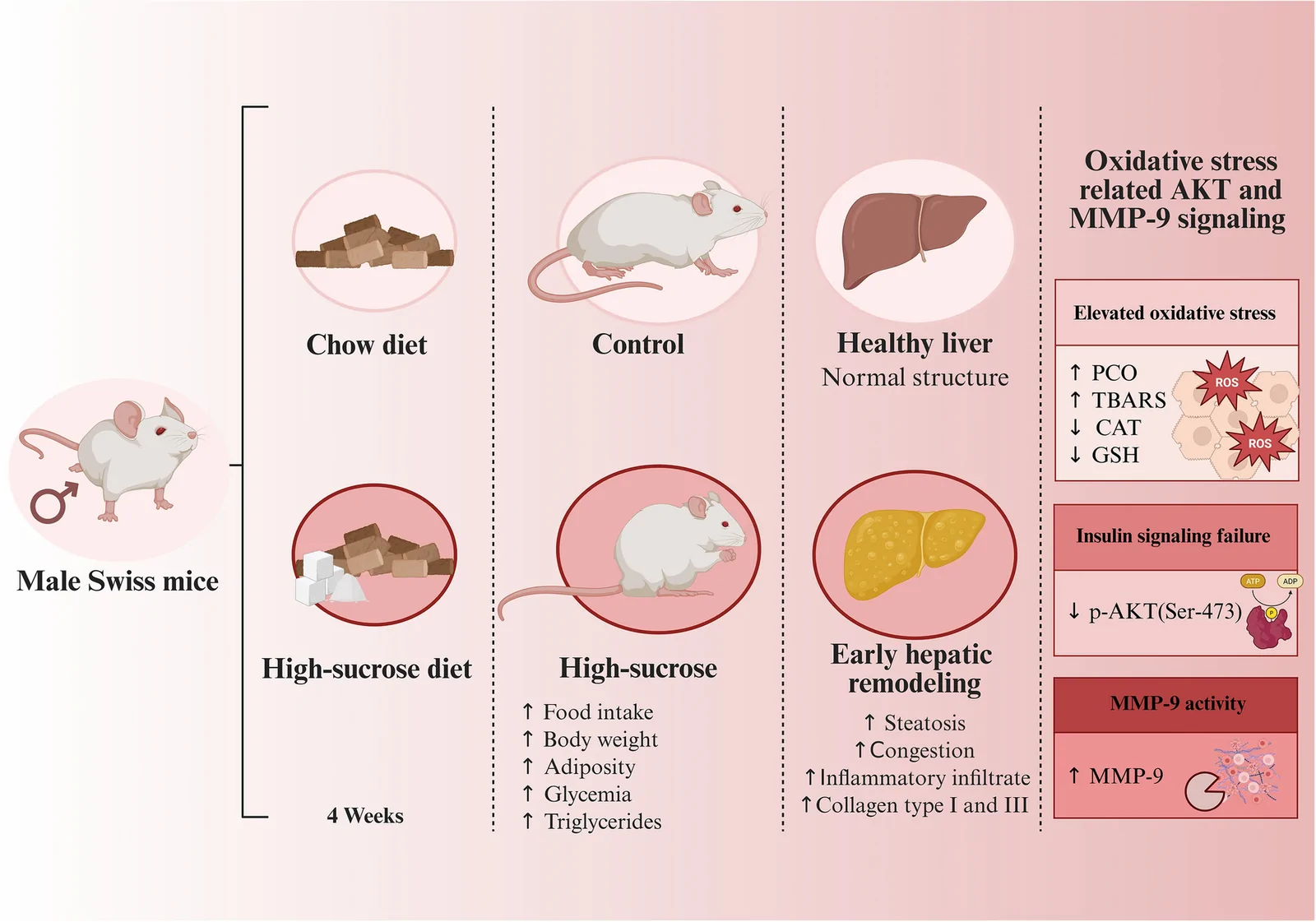

## Introduction

Excessive intake of a high-sucrose diet results in damage to biomolecules such as lipids, proteins, and nucleic acids, compromising cellular integrity and liver function primarily through increased oxidative stress. This redox imbalance is closely associated with the development of several liver pathologies, including steatosis, fibrosis and hepatocellular carcinoma (Sun et al. 2021; Yu et al. 2021). In addition, oxidative stress can modulate the expression and activity of specific enzymes, such as metalloproteinases (MMPs), particularly MMP-2 and MMP-9, which are implicated in liver damage and remodeling processes (Cabral-Pacheco et al. 2020; Aouey et al. 2024).

The MMPs are responsible for the remodeling and degradation of protein components belonging to the extracellular matrix. They are essential for physiological processes such as cell proliferation, migration, differentiation, angiogenesis, embryogenesis, wound healing, and even platelet aggregation (Hey and Linder 2024). However, deregulated activity of MMPs has been linked to a wide range of pathological conditions, including cardiovascular disease, kidney injury, cancer, inflammatory disorders, and liver pathologies (Bassiouni et al. 2021).

In the liver, the MMPs are directly involved in different stages of liver pathologies, including lesions, inflammation, and fibrosis (Cui et al. 2017). MMP-2 is widely expressed in various liver cells, especially hepatic stellate cells and Kupffer cells, and is frequently investigated in cases of liver fibrosis. Further, MMP-9 has been identified by some studies as a prognostic marker for liver cancer. Additionally, its activity is significantly increased in acute and fulminant liver failure (Geervliet et al. 2020). The MMP-2 and MMP-9 are activated by various factors, including inflammation and oxidative stress, and play a crucial role in pathological processes such as liver fibrosis and liver diseases (Sabir et al. 2023).

The expression and activity of MMPs are tightly regulated by transcription mechanisms, translation, and endogenous tissue of inhibitor matrix metalloproteinases (TIMPs) (Quintero-Fabián et al. 2019; Geervliet et al. 2020). Importantly, sucrose excess in the liver is related to the deregulation of insulin signaling, which can lead to insulin resistance (IR), influence MMP activity, and contribute to hepatic injury. Thus, this study aimed to evaluate the metabolic impact of a high-sucrose diet consumption in the MMP-2 and MMP-9 activities, collagen deposition, and histopathological changes in the liver of Swiss mice, through oxidative stress and insulin signaling disruption.

## Methodology

### Animals and diet

All experimental procedures in this study were conducted in accordance with the ethical guidelines for animal research established by the Brazilian National Council for the Control of Animal Experimentation (CONCEA) and approved by the Ethics Committee on Animal Use of the Federal University of Ouro Preto (protocol no. 7,422,050,723), as well as the *Principles of Laboratory Animal Care* published by the U.S. National Institutes of Health (NIH publication no. 86–23, revised 1985). Young male Swiss mice aged 21–35 days, corresponding to an early post-weaning/young developmental period, were used in this study. Initially, the male Swiss mice were provided with a balanced diet (NUVILAB CR1 – Nuvital-CR, Colombo, Brazil) and water ad libitum for 2 days for acclimatization. After this period, all animals were weighed and then randomly allocated to the two experimental groups, while ensuring a comparable distribution of initial body mass between groups. A total of 32 animals were assigned: control group (CON; *n* = 16), fed with the standard Nuvilab^®^ CR1 diet; and high-sucrose diet group (HS, *n* = 16), fed a diet containing 30% sucrose, composed of 40.44% Nuvilab^®^ chow, 40.44% Nestlé® Moça (Nestlé Brasil Ltda., São Paulo, Brazil) sweetened condensed milk, 8.57% refined sugar, and 10.55% water (de Deus et al. 2025). Throughout the 4-week experimental protocol, animals were housed under a 12:12 h light–dark cycle with free access to food and water. Environmental enrichment was provided using polyvinyl chloride (PVC) tubes. At the end, all 32 animals completed the experimental protocol and were anesthetized by inhalation of 3% isoflurane (Isoforine®, São Paulo, Brazil) for approximately 1 min and euthanized. Blood samples were collected in the morning at the time of euthanasia, without prior fasting, into 1.5 mL microtubes, centrifuged using a micro high-speed refrigerated centrifuge (VS-15000CFN II, Vision Scientific Co., Ltd., Daejeon, Republic of Korea), and the serum was stored at −80 °C for future analyses. The liver was immediately removed, weighed, transferred to cryotubes, snap-frozen in liquid nitrogen, and stored at −80 °C until further analysis. The reduction in the number of biological replicates varied between groups due to assay-specific technical limitations, such as hemolysis or insufficient tissue yield, which affected both groups independently. The exact number of animals for each endpoint is specified in the figure legends.

### Biochemical analyses

The biochemical parameters (glycemia, triglycerides, and total cholesterol) were determined in serum previously stored at −80 °C using colorimetric kits from Bioclin (Quibasa-Bioclin, Minas Gerais, Brazil). Serum glucose concentration was measured using the Glucose Monoreagent Kit (K082). The calculation was performed as (sample absorbance/standard absorbance) × 100. The serum total cholesterol concentration was measured using the Total Cholesterol Monoreagent kit (K083), using a similar method but with a multiplication factor of 200. For the determination of serum triglycerides concentration, the Triglycerides Monoreagent kit (K117) was used, with calculations following the same procedure as for glucose. The absorbance was measured using the colorimetric method with a microplate spectrophotometer (Biotek Epoch, Washington, WA) following the recommendations of the respective Bioclin kits. The absorbance was measured between 490 and 550 nm for glucose and between 490 and 540 nm for total cholesterol and triglycerides. All results were expressed in mg/dL.

### Serum markers of liver damage

Blood samples were centrifuged (VS-15000CFN II, Vision Scientific) to obtain serum, which was subsequently analyzed for biochemical indicators of hepatic injury. The assessments followed the protocols provided by the manufacturers of the commercial kits. The activities of aspartate aminotransferase (AST; K048-6) and alanine aminotransferase (ALT; K049-6) were quantified using reagents from Labtest Diagnóstica S.A. (Labtest Diagnóstica, Minas Gerais, Brazil). Absorbance was measured with a spectrophotometer (Biotek Epoch) at 340 nm, and the enzymatic activities were reported in U/mL.

### Superoxide dismutase and catalase activity and reduced glutathione concentration

The analysis of the antioxidant activity into the liver was performed through the assessment of superoxide dismutase (SOD) and catalase (CAT) activities and reduced glutathione (GSH) concentration. SOD was measured according to the method of Marklund and Marklund (1974) and data were expressed as units of SOD/mg protein (Marklund and Marklund 1974). CAT was measured according to the method of Beers and Sizer (1952) and data were expressed as mmol of H_2_O_2_ consumed/min/mg protein (Beers and Sizer 1952). The concentration of GSH was evaluated by analyzing sulfhydryl group oxidation with 5,5′-dithio-bis-(2-nitrobenzoic acid) (DTNB) (Sigma Aldrich, St. Louis, MO, USA), which forms a yellow-colored 5′-thio-2-nitrobenzoic (TNB), which was measured at 415 nm. The result was expressed as µg GSH/mg protein (Jones 2016).

### Oxidative stress biomarkers: protein carbonyl, thiobarbituric acid reactive substances concentration and thiol groups

Protein carbonyl groups (PCO) concentration was measured by monitoring the generation of 2,4-dinitrophenylhydrazone (DNPH) at 370 nm, after the reaction of the carbonyl groups in the oxidized proteins with DNPH (Levine et al. 1994). Liver samples were homogenized in 1.5 mL KPE (Potassium Phosphate Buffer with EDTA, Sigma Aldrich) using a polytron a D-160 homogenizer (DLAB Scientific Co., Ltd., Beijing, China). The homogenates were centrifuged (VS-15000CFN II, Vision Scientific) at 7.490 × g for 10 min at 4 °C, and the supernatants were used for analysis. The PCO concentration in the sample was estimated using the molar extinction coefficient of hydrazone (*ε* = 22.000 M^−1^ cm^−1^). The results were expressed in terms of nmol/mg protein.

Lipid peroxidation products were measured by the thiobarbituric acid (TBA) test (Kohn and Liversedge 1944). TBA reacts mainly with malondialdehyde, generating products (thiobarbituric acid reactive substances (TBARS)) whose levels were measured spectrophotometrically (535 nm) (Biotek Epoch). Liver samples were homogenized in 1 ml of 1.15% potassium chloride using a polytron (D-160 homogenizer, DLAB Scientific). The homogenates were centrifuged (VS-15000CFN II, Vision Scientific) at 7.490 × g for 10 min at 4 °C, and the supernatants were used for analysis. An analytical curve with 1,1',3,3'-tetramethoxypropane (Sigma Aldrich) was used as the standard. The results were expressed in terms of μmol/mg protein (Kohn and Liversedge 1944).

### Histopathological evaluations

The livers were harvested after 4 weeks of treatment, fixed in 10% neutral buffered formalin solution prepared with formaldehyde 37% pure inhibited solution (ACS Científica Química Fina Especializada Ltda., São Paulo, Brazil), anhydrous monobasic sodium phosphate (Dinâmica Química Contemporânea Ltda., São Paulo, Brazil), anhydrous dibasic sodium phosphate (Êxodo Científica, São Paulo, Brazil), and distilled water, with pH adjusted to 7.2, for approximately 72 h, then embedded in paraffin. The livers were processed using routine histological techniques for 4 μm thick paraffin sections, which were then mounted on glass slides. The histological sections were stained with hematoxylin and eosin (H&E) to evaluate steatosis and inflammatory infiltrate. For these quantifications, 30 random images of each animal were used at 40 × objective lens (numerical aperture (NA) 0.75; frame dimensions: 321 × 240 μm; total frame area: 77,040 μm^2^), obtained with a Leica DM5000B microscope with a digital camera (Leica DFC 300 FX; Leica Microsystems, Wetzlar, Germany) coupled to an activated RGB module and connected to the image capture software Leica Application Suite. Hepatic steatosis was analyzed using ImageJ software (version 1.51 k, National Institutes of Health, available at http://rsb.info.nih.gov/ij/) and a semiquantitative score system measured for evaluation on the percentage of hepatic lipid droplets. Steatosis, macrovesicular, microvesicular, and congestion was classified as of % hepatocytes affected: absent (0%), discrete (1–33%), moderate (34–66%), and intense (67–100%) (Carvalho et al. 2018). To determine the inflammatory pattern, the total number of cell nuclei present in each image, and the total number of cells, were quantified using Leica Qwin Plus software (Leica Microsystems), as previously described (Abreu et al. 2014). For the evaluation of fibrosis, Picrosirius Red staining was used to determine tissue collagen by optical microscopy in polarized light. A total of 20 random images were obtained of each animal at 10 × objective lens (numerical aperture (NA) 0.25; frame dimensions: 1283 × 958 μm; total frame area: 1,229,114 μm^2^). The deposition of collagen type I and III was measured in ImageJ software (Junqueira et al. 1979).

### Matrix metalloproteinase activities

The enzymatic activities of MMP-2 and MMP-9 in liver tissue were assessed through gelatin zymography following the method adapted from (Ceron et al. 2012; Martins et al. 2025). The liver homogenates (15 μg of protein) were subjected to a 10% sodium dodecyl-sulfate polyacrylamide gel electrophoresis (SDS-PAGE) containing a gelatin 1% electrophoresis system (BioRad®, Hercules, CA). Following electrophoresis, the gels were incubated for 1 h with a 2% Triton X-100 solution (Sigma-Aldrich) at room temperature. Then, the gels were incubated for 24 h at 37 °C in Tris–HCl buffer (Tris 50 mM, ZnCl_2_ 1 mM, pH 7.4) containing 10 mM CaCl_2_ (Sigma-Aldrich). Finally, the gels were stained for 1 h with 0.05% Coomassie Brilliant Blue G-250 (Sigma-Aldrich), followed by a destaining solution containing 30% methanol and 10% acetic acid (Alphatec, Rio de Janeiro, Brazil). The intensity of the lytic bands corresponding to MMPs was quantified by densitometric analysis using ImageJ software (version 1.51 k, National Institutes of Health, available at http://rsb.info.nih.gov/ij/). Results were expressed in arbitrary units (AU).

### Western blot analysis

Protein expression levels of p-AKT(Ser-473) and total AKT were assessed by western blot using the following specific antibodies: rabbit polyclonal (anti-phospho-AKT pSer473, #SAB4504331, Sigma-Aldrich, USA; and anti-AKT, #SAB4500799 Sigma-Aldrich, USA), and mouse monoclonal (anti-β-actin, sc-47778, Santa Cruz Biotechnology, USA). Liver tissue (100 mg) was homogenized in the RIPA lysis buffer (Sigma-Aldrich), followed by centrifugation (VS-15000CFN II, Vision Scientific) at 10.786 × g for 40 min at 4 °C. The total protein concentration was quantified using the Lowry method (Lowry et al. 1951). Protein extracts (50 μg) were separated via SDS-PAGE 10% and transferred to nitrocellulose membranes (BioRad®). Membranes were incubated overnight with primary antibodies (1:1000 for anti-AKT, anti-p-AKT, and anti-β-actin), followed by secondary antibody incubation (1:5000 anti-rabbit, #7074S, Cell Signaling Technology, USA; and 1:5000 anti-mouse #7076S, Cell Signaling Technology, USA). Antibody specificity was validated by the manufacturers, and validation data are provided in the technical datasheets supplied by Sigma-Aldrich, Santa Cruz Biotechnology, and Cell Signaling Technology. Detection was carried out using the ECL system (Sigma-Aldrich). The β-actin served as the endogenous control. Protein expression levels were quantified using ImageJ software (version 1.51 k, National Institutes of Health, available at http://rsb.info.nih.gov/ij/).

Western blotting was performed using eight liver samples per group. The analysis was conducted only in samples with sufficient protein yield, adequate sample integrity, and reliable detection of both the target protein and endogenous control. Samples were not excluded on the basis of the direction or magnitude of the results, but only according to sample availability and technical suitability for the assay.

### Statistical analyses

The data were expressed as mean ± standard deviation. Statistical analyses were performed using GraphPad Prism version 8.0.2 software (Prism Software, Irvine, CA). The Shapiro–Wilk test was used to assess the normality standards. The mean comparison test (Mann–Whitney or Student's *t*-test) was used to compare differences between means. Parametric results have been presented as mean ± standard deviation, and nonparametric results as median and percentiles. Differences with *p* ≤ 0.05 were considered significant. The number of biological replicates varied among assays due to sample availability, insufficient serum or tissue volume, protein yield, and/or technical limitations during sample processing and analysis. Samples were excluded only when unavailable or technically unsuitable for a given assay, and not on the basis of the direction or magnitude of the results. The exact *n* used for each parameter is indicated in the corresponding figure legends.

## Results

### High-sucrose diet increases body mass, food intake, and adipose tissue accumulation in Swiss mice

**Fig. 1:**
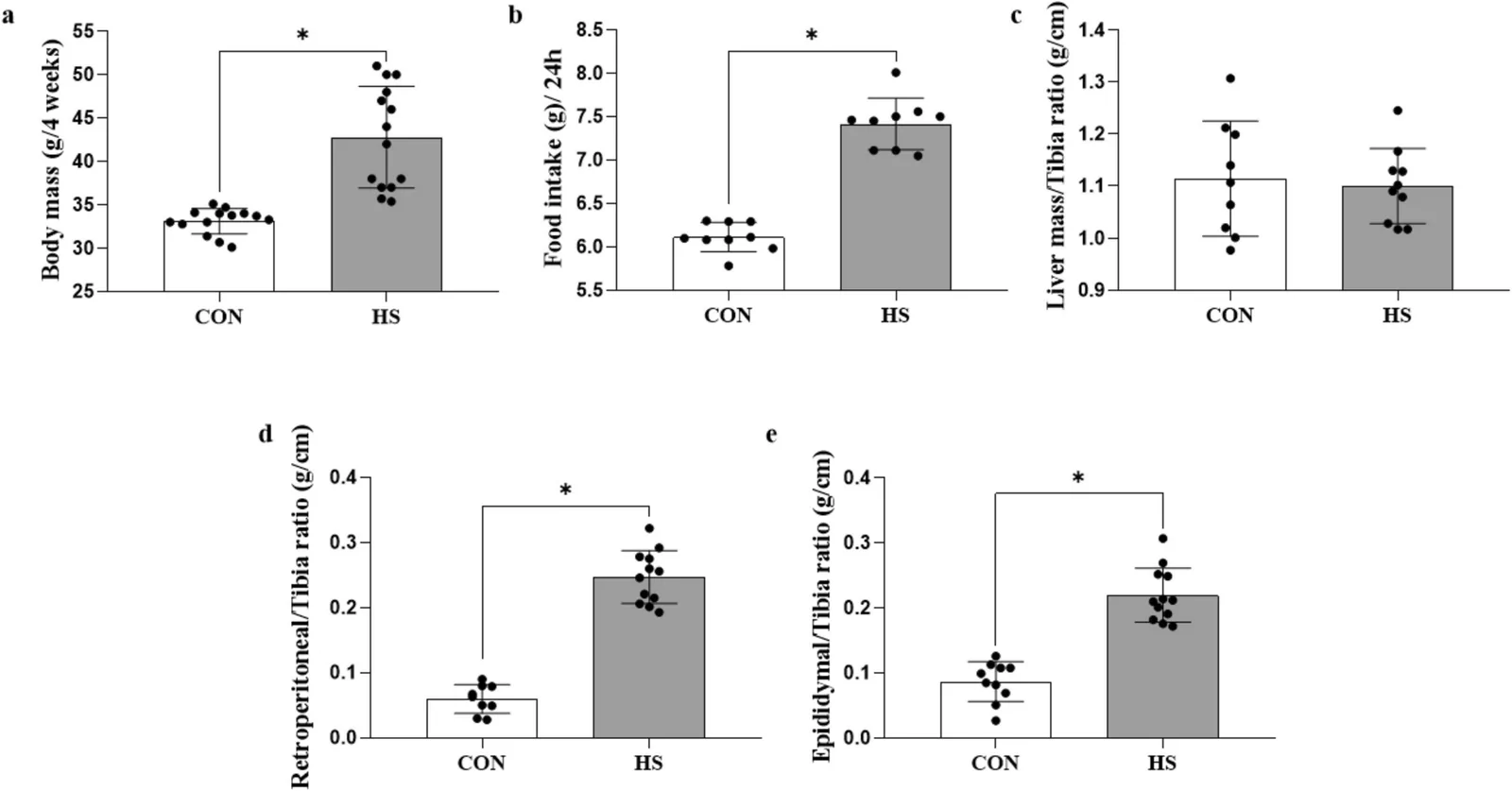
Effects of a high-sucrose diet on body mass, food intake, liver mass, and adipose tissue depots in Swiss mice. **a.** Body mass (g/4 weeks): CON, *n* = 14; HS, *n* = 14. **b.** Food intake (g/24 h): CON, *n* = 9; HS, *n* = 9. **c.** Liver mass normalized to tibia length (g/cm): CON, *n* = 9; HS, *n* = 10. **d.** Retroperitoneal (g/cm tibia): CON, *n* = 9; HS, *n* = 12. **e.** Epididymal (g/ cm tibia): CON, *n* = 10; HS, *n* = 12. Data were analyzed using the Shapiro–Wilk normality test followed by an unpaired Student’s *t*-test. Results are represented as mean ± standard deviation. Differences between groups were considered significant with *p* ≤ 0.05. *Indicates significant difference versus CON

To investigate the systemic effects of high-sucrose intake, we evaluated physiological parameters (body mass and food intake), tissue weights (liver, retroperitoneal, and epididymal fat depots), and biochemical markers (glycemia, triglycerides, and total cholesterol) in male Swiss mice after 4 weeks of dietary intervention. After 4 weeks of dietary intervention, the HS group showed a significant increase in body mass (42.79 ± 5.86 g, *p* < 0.0001) compared with the control group (32.12 ± 1.46 g) (Fig. 1a).

Food intake also increased in animals fed the high-sucrose diet (7.41 ± 0.29 g/24 h, *p* < 0.0001) compared with the control group (6.11 ± 0.16 g/24 h) (Fig. 1b). However, liver mass remained unchanged between groups (1.10 ± 0.07 versus 1.11 ± 0.11 g/cm tibia for HS and CON, respectively; *p* = 0.7483) (Fig. 1c). Conversely, adipose tissue depots were markedly affected by sucrose intake. The retroperitoneal fat pad was significantly higher in the HS group (0.247 ± 0.04 g/cm tibia, *p* < 0.0001) compared with CON (0.059 ± 0.02 g/cm tibia). Similarly, epididymal fat mass increased in HS animals (0.219 ± 0.04 g/cm tibia, *p* < 0.0001) compared with controls (0.086 ± 0.03 g/cm tibia) (Fig. 1d and e).

CON: control group; HS: high-sucrose diet group

### High-sucrose diet increases biochemical parameters in Swiss mice

**Fig. 2:**
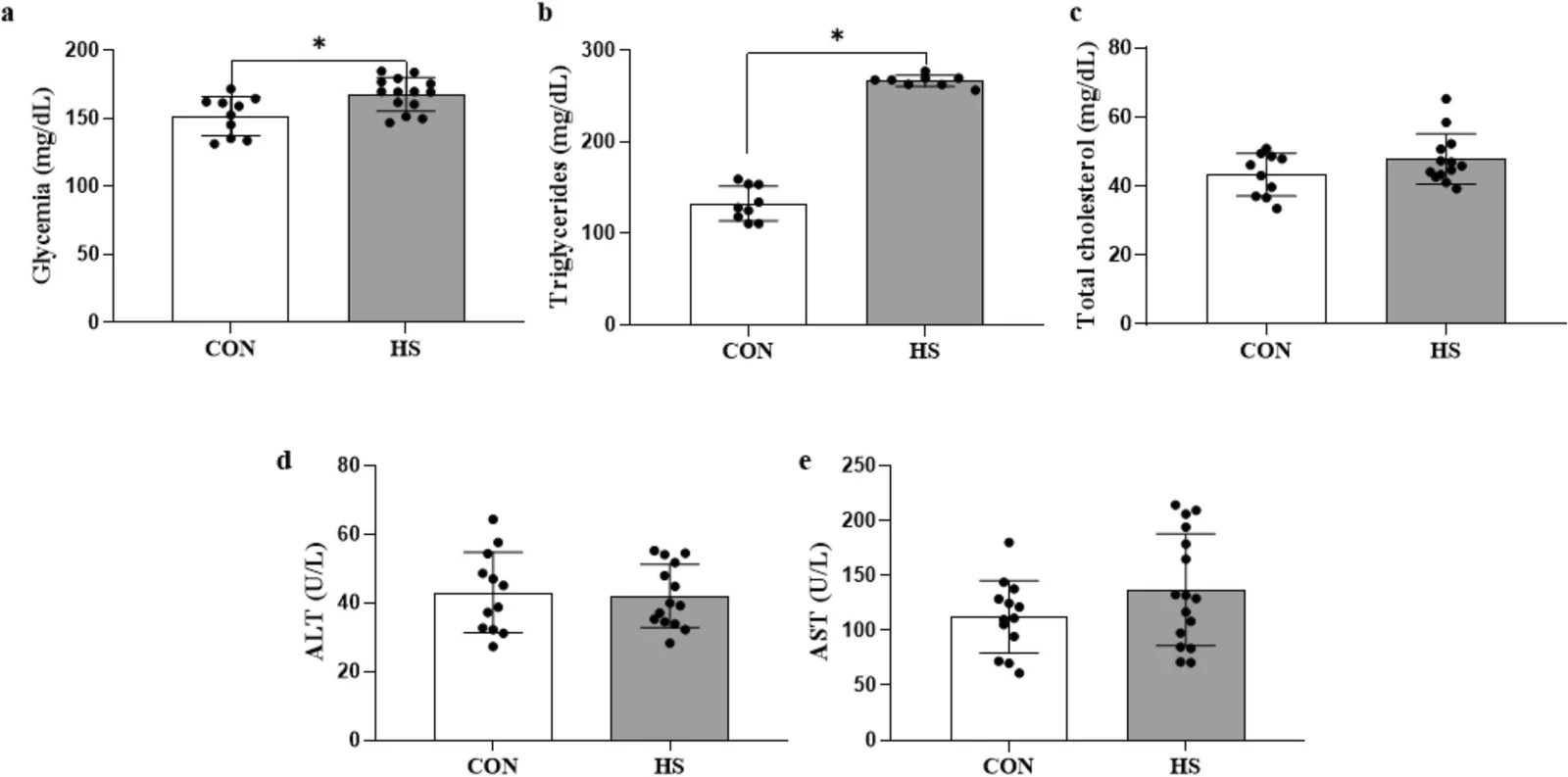
Effects of a high-sucrose diet on biochemical parameters in Swiss mice. **a.** Glycemia (mg/dL): CON, *n* = 10; HS, *n* = 14. **b.** Triglycerides (mg/dL): CON, *n* = 9; HS, *n* = 8. **c.** Total cholesterol (mg/dL): CON, *n* = 10; HS, *n* = 13. **d.** ALT (U/L): CON, *n* = 12; HS, *n* = 14. **e.** AST (U/L): CON, *n* = 13; HS, n = 16. Data were analyzed using the Shapiro–Wilk normality test followed by an unpaired Student’s *t*-test. Results are represented as mean ± standard deviation. Differences between groups were considered significant with *p* ≤ 0.05. *Indicates significant difference versus CON

In terms of biochemical parameters, the HS group exhibited elevated glycemia (167.6 ± 12.2 mg/dL, *p* < 0.0075) compared with CON (151.6 ± 14.4 mg/dL) (Fig. 2a). Triglycerides levels were markedly increased in HS animals (266.7 ± 6.10 mg/dL, *p* < 0.0001) compared with the control group (132.5 ± 18.85 mg/dL) (Fig. 2b). In contrast, total cholesterol concentration showed no significant difference between groups (47.79 ± 7.29 versus 43.27 ± 6.19 mg/dL for HS and CON, respectively, *p* = 0.1314) (Fig. 2c).

Regarding hepatic enzymes, ALT and AST showed no significant difference between groups. ALT levels in the HS group (42.06 ± 9.19 U/L, *p* = 0.8135) did not differ from those in the compared with CON (43.04 ± 11.72 U/L). Similarly, AST levels in HS animals (137.1 ± 50.91 U/L, *p* = 0.1421) did not differ from those in compared with control animals (112.3 ± 33.07 U/L) (Fig. 2d and e).

CON: control group; HS: high-sucrose diet group; ALT: alanine aminotransferase; AST: aspartate aminotransferase

### High-sucrose diet alters antioxidant defenses and increases oxidative stress biomarkers in the liver of Swiss mice

**Fig. 3:**
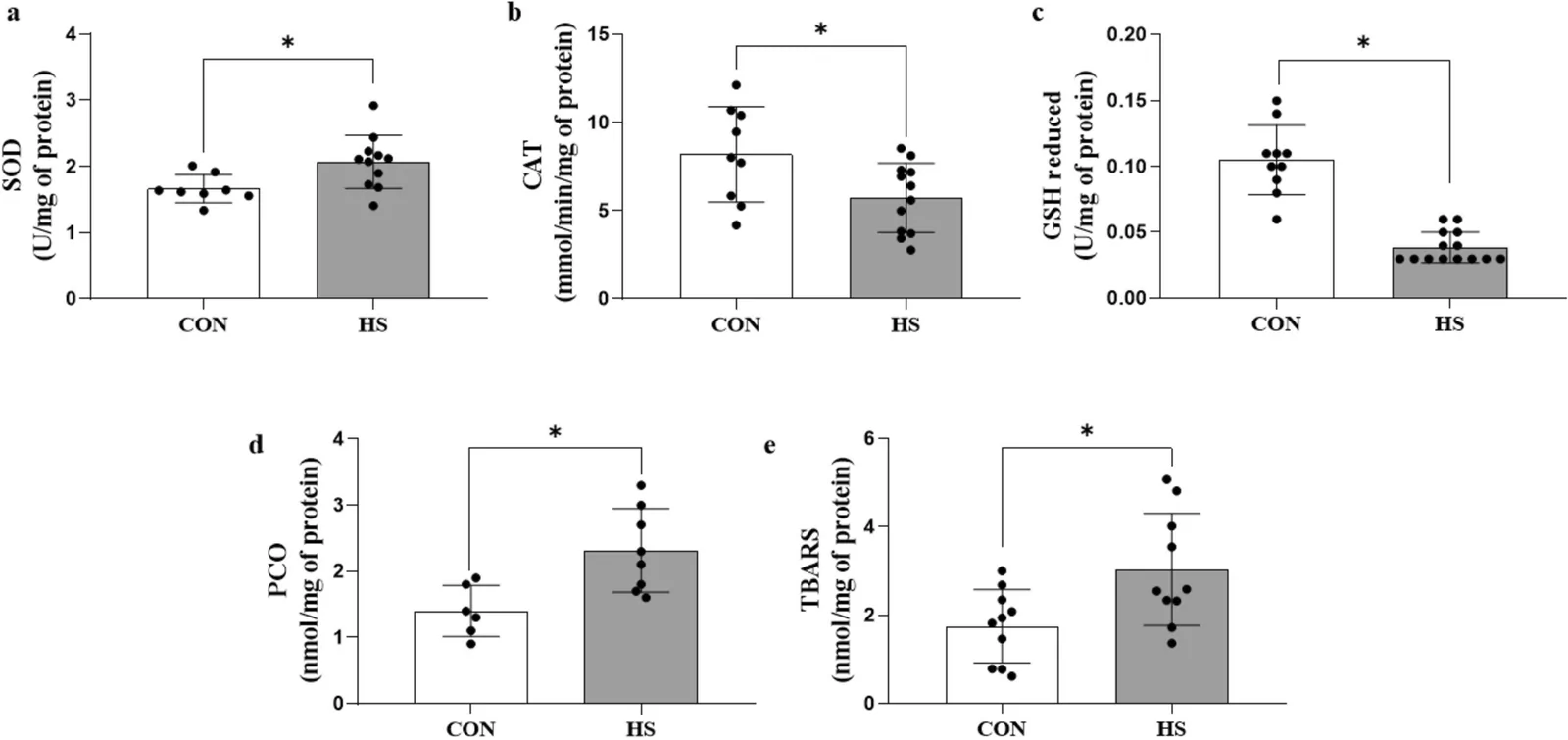
Effect of a high-sucrose diet on antioxidant enzyme activities and oxidative stress markers in the liver of Swiss mice.** a**. SOD activity (U/mg protein): CON, *n* = 8; HS, *n* = 11. **b.** CAT activity (U/mg protein): CON, *n* = 9; HS, *n* = 12. **c.** Reduced GSH levels (U/mg protein): CON, *n* = 10; HS, *n* = 14. **d.** PCO (nmol/mg protein): CON, *n* = 6; HS, *n* = 8. **e.** TBARS (nmol/L): CON, *n* = 10; HS, *n* = 10. Data were analyzed using the Shapiro–Wilk normality test followed by an unpaired Student’s *t*-test. Results represented as mean ± standard deviation. Differences between groups were considered with *p* ≤ 0.05. *Indicates significant difference versus CON

After 4 weeks of HS consumption, an increase in SOD activity was observed in the liver of HS group mice (2.07 ± 0.40 U/mg protein, *p* = 0.019) compared with the control group (1.67 ± 0.21 U/mg protein) (Fig. 3a). In contrast, there was a reduction in CAT activity in the HS group (5.73 ± 1.96 U/mg protein, *p* = 0.026) compared with control (8.19 ± 2.70 U/mg protein) (Fig. 3b). Additionally, the HS group showed a marked decrease in reduced GSH levels (0.038 ± 0.011 µg/mg protein, *p* < 0.0001) compared with the control group (0.105 ± 0.026 µg/mg protein), indicating impaired hepatic antioxidant capacity (Fig. 3c).

Regarding oxidative damage, the high-sucrose diet significantly increased protein oxidation, as evidenced by the elevated levels of PCO in the HS group (2.31 ± 0.631 nmol/mg protein, *p* = 0.0091) compared with the control group (1.40 ± 0.389 nmol/mg protein) (Fig. 3d). Similarly, lipid peroxidation was increased, as shown by higher concentrations of TBARS in the HS group (3.033 ± 1.27 nmol/mg protein, *p* = 0.0155) compared with the control (1.750 ± 0.83 nmol/mg protein) (Fig. 3e).

CON: control group; HS: high-sucrose diet group; SOD: superoxide dismutase; CAT: catalase; GSH: reduced glutathione; PCO: protein carbonyl groups; TBARS: thiobarbituric acid reactive substances

### High-sucrose diet increases hepatic steatosis and induces congestive hepatopathy in Swiss mice

**Fig. 4:**
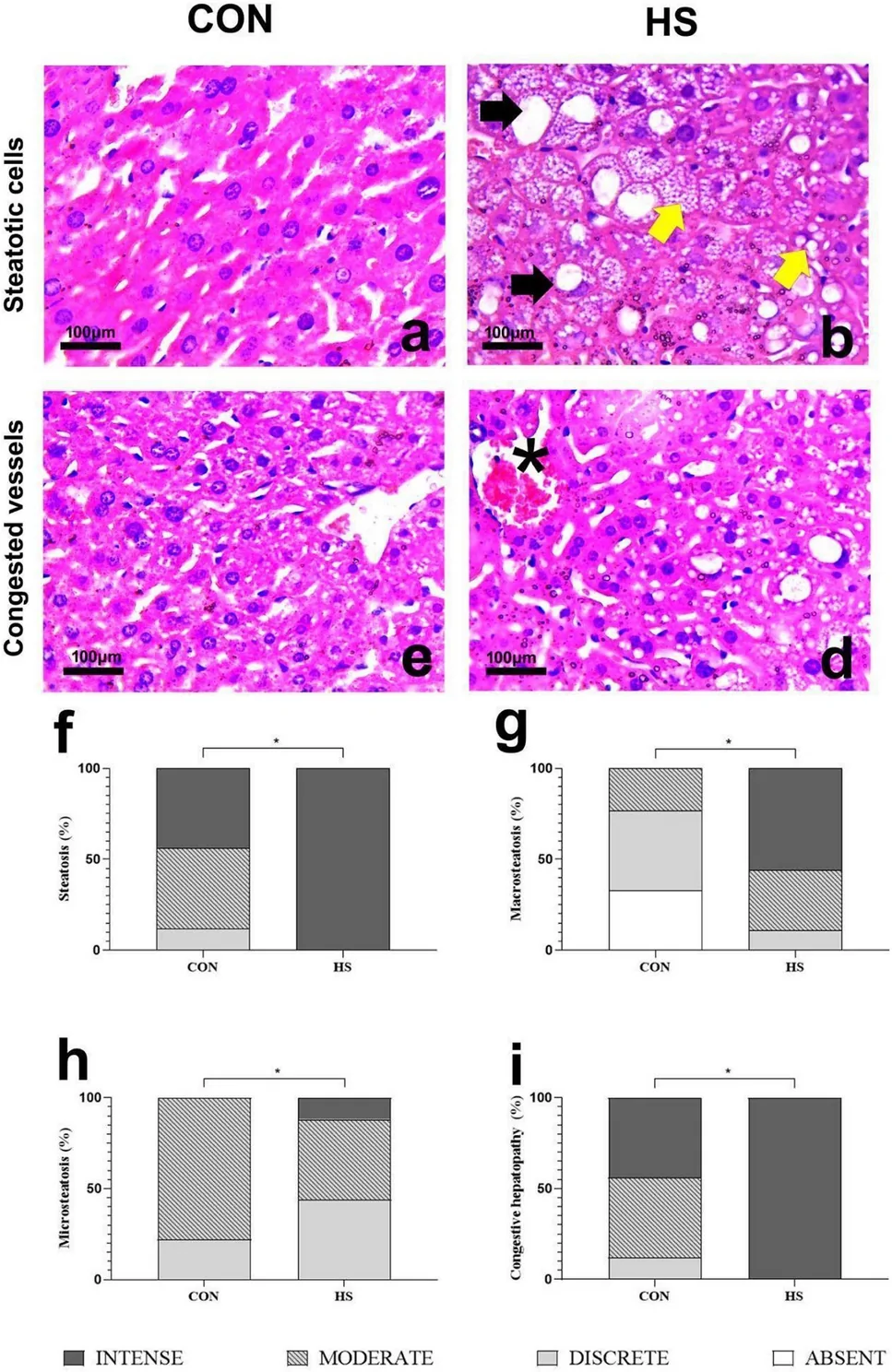
Effect of a high-sucrose diet on liver steatosis, macrosteatosis, microsteatosis, and congestive hepatopathy in Swiss mice. Representative histological images of the experimental groups **a–d** at a 40 × objective (400 × magnification) obtained with a Leica DM5000B microscope with a DFC 300 FX camera, and quantitative analyses of experimental groups. Scale bars: 100 µm. Sections were stained with H&E, and 30 random images per animal were analyzed using ImageJ software. A semiquantitative score system was used to determine the percentage of lipid droplet deposition. Steatosis, macrovesicular steatosis, microvesicular steatosis, and congestion were classified according to the percentage of hepatocytes affected and congested vessels: absent (0%), discrete (1–33%), moderate (34–66%), and intense (67–100%). **a–b.** Steatotic cells and **c–d.** congestive vessels **f.** Steatosis (%): CON, *n* = 9; HS, *n* = 9. **g.** Macrosteatosis (%): CON, *n* = 9; HS, *n* = 9. **h.** Microsteatosis (%): CON, *n* = 9; HS, *n* = 9. **i.** Congestive hepatopathy (%): CON, *n* = 9; HS, *n* = 9. Data were analyzed using a chi-squared contingency test. Results are represented as frequencies. Differences between groups were considered significant with *p* ≤ 0.05. *Indicates significant difference versus CON. **b.** Black arrows represent macrovesicular steatosis and yellow arrows represent microvesicular steatosis. **d.** The congestive vessel is indicated by an asterisk (*). CON: control group; HS: high-sucrose diet group

The high-sucrose diet markedly affected hepatic morphology. All animals in the HS group (100%) exhibited intense steatosis, whereas in the control group, steatosis was distributed as follows: 12% discrete, 44% moderate, and 44% severe (Fig. 4a). Regarding macrosteatosis, the HS group showed 11% discrete, 33% moderate, and 56% intense patterns. In contrast, control animals showed 33% absent, 44% discrete, and 23% moderate (Fig. 4b). A similar pattern was observed for microsteatosis. The HS group showed 44% discrete, 44% moderate, and 12% intense, whereas control animals showed 22% discrete and 78% moderate microsteatosis (Fig. 4c). In addition, the high-sucrose diet promoted congestive hepatopathy in the HS group (11% moderate and 89% intense) compared with control animals (56% discrete, 33% moderate, and 11% intense) (Fig. 4d).

### High-sucrose diet increases inflammatory infiltrates and collagen deposition in the liver of Swiss mice

**Fig. 5:**
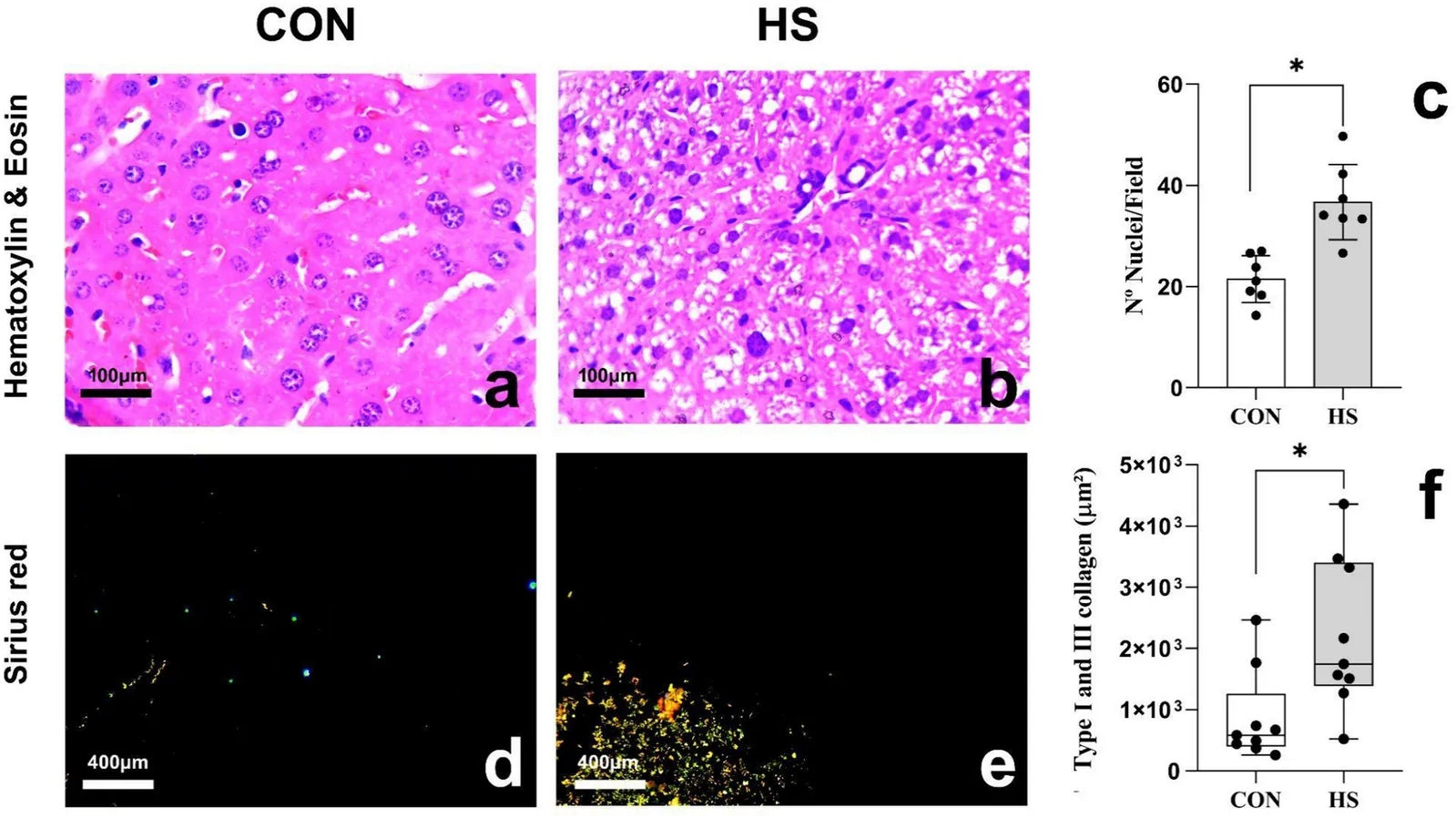
Effect of a high-sucrose diet on hepatic inflammatory cell infiltration and histological detection of collagen types I and III in the liver tissue. Sections were stained with H&E, and images were obtained at a 40 × objective (400 × magnification) using a Leica BM5000 microscope with a DFC 300 FX camera. Nuclei were counted manually in 30 random fields per animal using Leica Qwin Plus software. **a–b.** Representative liver sections stained with H&E in control and HS groups. Scale bars: 100 µm. **b.** Quantification of hepatic inflammatory infiltrates (nuclei/field) in the experimental groups, CON, *n* = 7; HS, *n* = 7. **d–e.** Representative sections stained with Sirius Red to assess collagen type I and III deposition in control and HS. Scale bars: 400 µm. **f.** Quantification of collagen type I and III (μm^2^) in the experimental groups, CON, *n* = 9; HS, *n* = 9. Data were analyzed using the Shapiro–Wilk normality test, unpaired Student’s *t*-test, Mann–Whitney test, and are represented as mean ± standard deviation. Differences between groups were considered with *p* ≤ 0.05. *Denotes comparison with CON. CON: control group; HS: high-sucrose diet group

Inflammatory cells increased in the HS group (36.71 ± 7.42 nuclei, *p* < 0.001) compared with the control group (21.46 ± 4.64 nuclei) (Fig. 5a). The high-sucrose diet promoted an increase in types I and III collagen fibers per field (1747 (1392–4359) µm^2^, *p* < 0.05 versus CON: 587.3 (406.7–2466) µm^2^) (Fig. 5b).

### High-sucrose diet increases MMP-9 activity in the liver of Swiss mice after 4 weeks

**Fig. 6:**
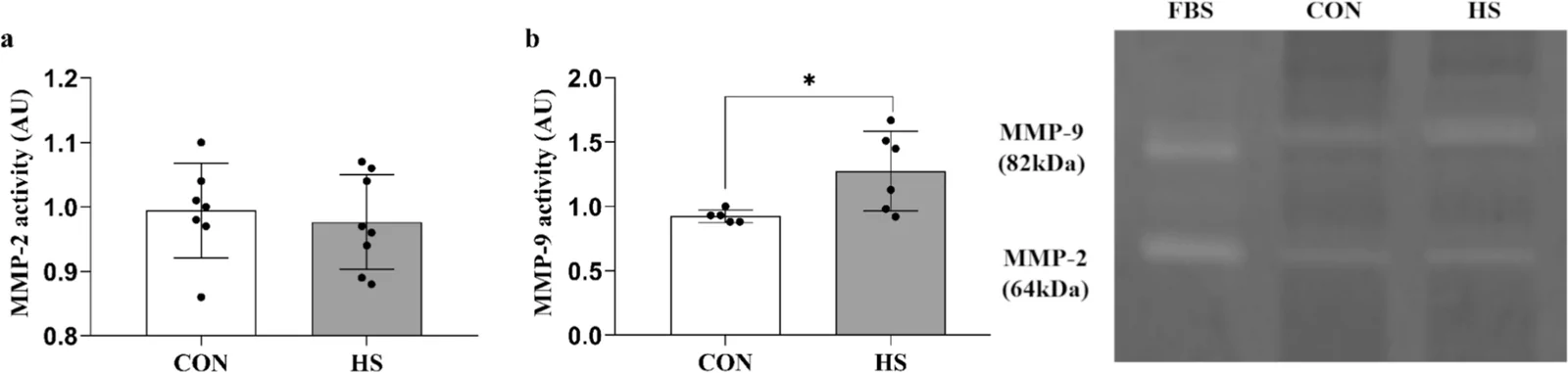
Effect of a high-sucrose diet on liver MMP-2 and MMP-9 activities determined by gelatin zymography. Liver homogenates (15 μg protein) were loaded into gelatin SDS-PAGE gels. Band intensities were quantified by densitometry using ImageJ software. **a.** MMP-2 activity (AU): CON, *n* = 7; HS, *n* = 8; **b.** MMP-9 activity (AU): CON, *n* = 5; HS, *n* = 6. Data were analyzed using the Shapiro–Wilk normality test and unpaired Student’s *t*-test and are represented as mean ± standard deviation. Differences between groups were considered with *p* ≤ 0.05. *Denotes comparison with CON

STD: standard; FBS: fetal bovine serum; MMPs: matrix metalloproteinases; CON: control group; HS: high-sucrose diet group

The high-sucrose diet did not alter MMP-2 activity in the liver (0.976 ± 0.073 AU, *p* = 0.6440) compared with the control group (0.994 ± 0.073 AU) (Fig. 6a). MMP-9 activity was increased in the HS group (1.277 ± 0.308 AU, *p* = 0.0335) compared with the control group (0.924 ± 0.049 AU) (Fig. 6b).

### High-sucrose diet decreases AKT phosphorylation in the liver of Swiss mice after 4 weeks

**Fig. 7:**
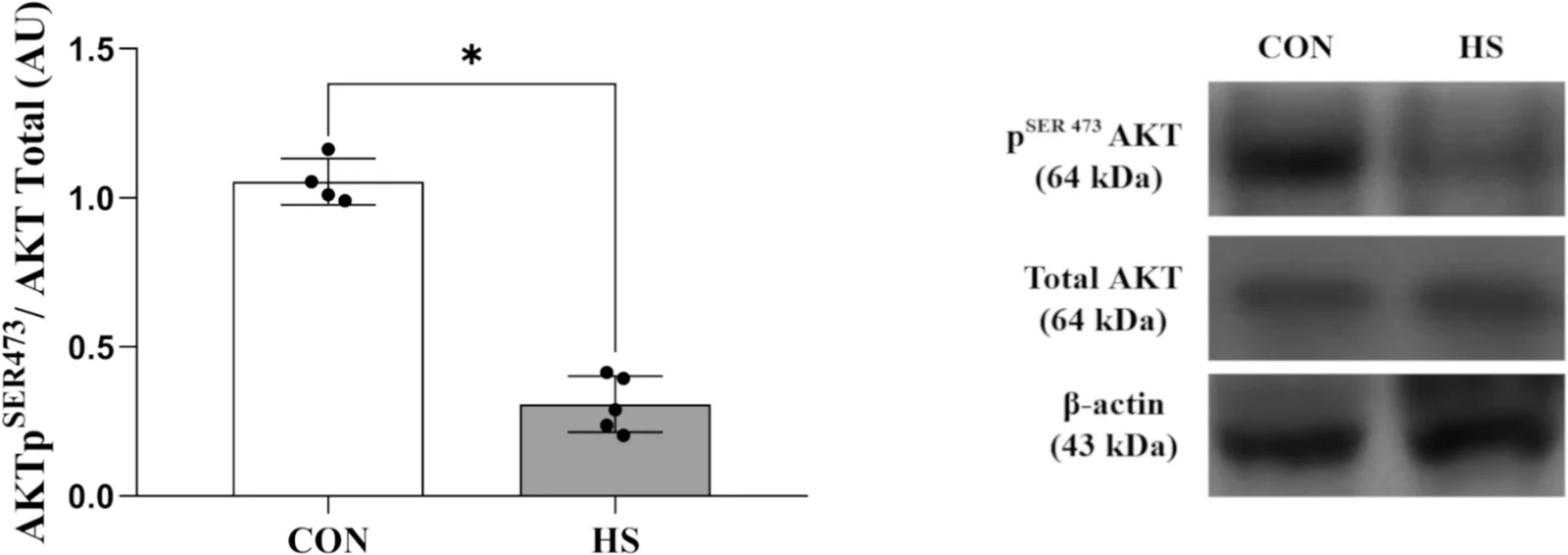
Effect of a high-sucrose diet on liver AKT ser-473 phosphorylation determined by western blotting. Band intensities were quantified by densitometry using ImageJ software. CON, *n* = 8; HS, *n* = 8. Data were analyzed using the Shapiro–Wilk normality test and unpaired Student’s *t*-test and are represented as mean ± standard deviation. Differences between groups were considered with *p* ≤ 0.05. *Denotes comparison with CON. CON: control group; HS: high-sucrose diet group

The high-sucrose diet decreased the expression of phosphorylated AKT at Ser-473 residue, as determined by Western blot densitometry, in the HS group (0.307 ± 0.093 AU, *p* < 0.0001) compared with the control group (1.054 ± 0.077 AU) (Fig. 7).

## Discussion

Carbohydrates play a crucial role in the body, providing the energy necessary for maintaining cellular homeostasis. They are important components in both human and rodent diets (Witek et al. 2022). However, excessive intake of sucrose-rich diets can have harmful effects on cellular homeostasis, contributing to the development of metabolic imbalances (Stanhope 2012; Fisher et al. 2024). In the present study, 4 weeks of a high-sucrose diet intake was sufficient to induce significant metabolic, oxidative, and molecular alterations that negatively impacted liver structure remodeling in male Swiss mice, reinforcing the deleterious impact of excessive dietary sugar intake even over a relatively short period. The 4-week dietary protocol was selected to evaluate early hepatic responses to excessive sucrose intake. Although longer protocols using high-sugar or high-fat/high-sugar diets are frequently used to investigate chronic metabolic alterations, short-term protocols have also demonstrated diet-induced metabolic, inflammatory, and tissue changes in mice (Khan et al. 2020; Li et al. 2024).

Animals fed the high-sucrose diet exhibited significant increases in body mass and food intake, accompanied by pronounced expansion of retroperitoneal and epididymal adipose tissue depots. These findings suggest that excess sucrose intake promotes positive energy balance and lipid accumulation, contributing to increased adiposity and body mass gain. Similar findings were reported by França et al. (2020), who observed that post-weaning male Swiss mice exposed to a high-sucrose diet exhibited increased body mass, hypertriglyceridemia, peripheral insulin resistance, and hepatic steatosis (França et al. 2020). Collectively, these data support the notion that excessive sugar consumption disrupts energy homeostasis by favoring lipid storage and adipose tissue expansion, ultimately contributing to metabolic imbalance (de Queiroz et al. 2014; França et al. 2020).

Metabolic alterations were further suggested by a modest but significant increase in serum glucose levels in animals fed the high-sucrose diet. Since blood samples were collected in the morning at the time of euthanasia without prior fasting, these values should be interpreted as non-fasting serum glucose levels. Therefore, serum glucose alone should not be interpreted as diagnostic evidence of type 2 diabetes. Together with the marked increase in triglycerides, adipose tissue accumulation, and reduced hepatic AKT phosphorylation, these findings suggest early diet-induced metabolic impairment. Hyperglycemia has been consistently reported in rodents exposed to sucrose-rich diets, particularly after prolonged dietary interventions (Żebrowska et al. 2020; de Deus et al. 2025). In parallel, the marked increase in circulating triglycerides is consistent with enhanced hepatic lipogenesis and subsequent storage of triacylglycerols in adipose depots. These alterations reflect impaired glucose and lipid metabolism and reinforce the link between excessive sucrose intake and dyslipidemia, as previously described in experimental models of carbohydrate-induced metabolic dysfunction (de Deus et al. 2025).

Regarding hepatic antioxidant defenses, 4 weeks of high-sucrose consumption resulted in increased superoxide dismutase activity. This response may indicate an early compensatory mechanism aimed at counteracting increased reactive oxygen species (ROS) production. Such an adaptive increase in SOD activity has been described as an initial attempt to maintain redox homeostasis under conditions of metabolic stress. However, this response was accompanied by a reduction in catalase (CAT) activity and a marked depletion of reduced glutathione (GSH), indicating an overall impairment of hepatic antioxidant capacity. These findings suggest that, despite an initial upregulation of SOD, the antioxidant system becomes unbalanced, favoring oxidative stress. In line with this interpretation, Oliveira et al. (2020) reported reduced SOD activity after longer periods of sucrose exposure, while Zhang et al. (2021) demonstrated decreases in both SOD and CAT activities following prolonged consumption of high-fat and high-sugar diets (Oliveira et al. 2020; Zhang et al. 2021).

Consistent with the impairment of antioxidant defenses, the high-sucrose diet promoted significant oxidative damage in the liver, as evidenced by increased protein carbonylation (PCO) and thiobarbituric acid reactive substances (TBARS). These markers reflect oxidative modifications of macromolecules and indicate sustained redox imbalance. Similar increases in TBARS and PCO levels have been reported in rodents fed carbohydrate- and fat-rich diets, reinforcing the role of excessive sugar intake in promoting oxidative stress and hepatic injury (Żebrowska et al. 2020). These findings collectively indicate that a high-sucrose diet impairs hepatic antioxidant defenses while promoting oxidative stress and macromolecular damage.

Histopathological analysis further revealed that the high-sucrose diet markedly affected liver morphology, including elevated steatosis (Oliveira et al. 2020). Animals in the high-sucrose group exhibited intense hepatic steatosis, accompanied by both macro- and microvesicular lipid accumulation, as well as pronounced congestive hepatopathy. These alterations occurred despite the absence of significant changes in circulating ALT and AST levels, suggesting that the observed damage represents an early stage of hepatic dysfunction, prior to overt hepatocellular injury. Similar dissociations between histological liver damage and serum transaminase levels have been reported in early stages of diet-induced fatty liver disease, highlighting the limited sensitivity of ALT and AST as early biomarkers of hepatic injury (Softic et al. 2017; Sun et al. 2021). This dissociation underscores the sensitivity of morphological and molecular markers, suggesting that excessive sucrose intake causes steatosis that precedes changes in conventional aminotransferases.

In addition to steatosis, the high-sucrose diet increased inflammatory cell infiltration and collagen deposition in the liver, suggesting the initiation of tissue remodeling processes. Excessive collagen accumulation, particularly type III fibers, reflects early fibrogenic responses and is tightly associated with oxidative-stress-driven inflammation and activation of hepatic stellate cells (Yu et al. 2021). Experimental studies have demonstrated that diets rich in simple sugars are sufficient to induce hepatic inflammation and early fibrotic remodeling, even in the absence of advanced liver injury (Yu et al. 2021; Żebrowska et al. 2020; Softic et al. 2017). Together, these findings suggest that short-term sucrose overload can activate inflammatory and profibrotic pathways, potentially predisposing the liver to progressive structural damage if dietary exposure is sustained.

MMP analysis demonstrated increased MMP-9 activity in the liver of animals fed the high-sucrose diet, while MMP-2 activity remained unchanged. MMP-9 activation has been strongly associated with oxidative stress and inflammatory signaling pathways. Tsai et al. (2013) also observed an increase in MMP-9 activity in tendon cells exposed to high glucose concentrations, whereas Chi et al. (2014) reported an increase in MMP-2 activity in fibroblast cell cultures incubated in glucose-rich medium. Additional in vivo studies have shown that high-sugar diets upregulate MMP-9 expression and activity in metabolic tissues, linking excessive carbohydrate intake to extracellular matrix remodeling and inflammation (Tsai et al. 2013; Chi et al. 2014).

Finally, excessive intake of carbohydrates is directly linked to disruption of the classic insulin signaling pathway, the PI3K/AKT. In the present study, 4 weeks of high-sucrose diet intake significantly reduced AKT phosphorylation at serine 473 in the liver, suggesting that this hormone lost its ability to act and regulate hepatic metabolism. Similar reductions in AKT phosphorylation have been reported in metabolic tissues of rodents exposed to sucrose-rich or high-fat diets, highlighting the vulnerability of insulin signaling pathways to dietary excess (Oliveira et al. 2020; Softic et al. 2017). These findings reinforce the concept that excessive sugar intake, even over a short duration, is sufficient to disrupt metabolic homeostasis.

## Conclusions

The results suggest that a 4-week high-sucrose diet consumption disrupts hepatic redox homeostasis and insulin signaling by reduced AKT phosphorylation, leading to steatosis, elevated MMP-9 activity, and collagen deposition in the liver of Swiss mice. These findings partially support the hypothesis that excess sucrose promotes oxidative stress and MMP-9-mediated fibrogenic process.

## Study limitations

This study has limitations that should be considered when interpreting the findings. Although 32 animals were initially allocated equally to the 2 experimental groups, with 16 animals per group, the number of biological replicates varied across analyses. This variation resulted from the unavailability of some samples or from insufficient sample volume, tissue quantity, protein yield, or technical quality for specific assays. Additional factors included hemolysis and limitations encountered during biochemical and histological processing, gelatin zymography, or western blotting. Samples were excluded solely when unavailable or technically unsuitable for the respective assay and not according to the direction, magnitude, or statistical significance of the results. To improve transparency, the exact number of animals included in each analysis is reported in the corresponding figure legends.

## Data Availability

The data supporting the findings of this study are available upon request to the corresponding author: 10.5281/zenodo.18567165 and 10.5281/zenodo.20529450.
